# ICOS regulates ILC2s in asthma

**DOI:** 10.18632/oncotarget.5245

**Published:** 2015-08-22

**Authors:** Hadi Maazi, Omid Akbari

**Affiliations:** Department of Molecular Microbiology and Immunology, Keck School of Medicine of University of southern California, Los Angeles, CA, USA

**Keywords:** innate lymphoid cells, ILC2s, asthma, costimulatory molecules

Asthma is a chronic inflammation of the airways leading to airway hyperreactivity and bronchoconstriction in response to allergens as well as unspecific stimuli such as cold air [1]. Classically, the key player cells in the pathogenesis of asthma are known to be T helper cells subsets including Th2, Th9 and Th17 as well as iNK T cells and dendritic cells [1–3]. An important aspect of the classical model of the pathogenesis of asthma is the requirement for T cells and antigen specificity. Discovery of type 2 innate lymphoid cells (ILC2s) has substantially changed our understanding of the pathogenesis of asthma. ILC2s are a newly identified immune cells distinct from all known immune cells including T and B cells, dendritic cells, macrophages and granulocytes [4]. These new players in the pathogenesis of asthma can rapidly react to stimuli such as IL-25, IL-33 and TSLP and produce large amounts of Th2 cytokines, IL-5, IL-13 and the Th9 cytokine, IL-9 causing airway inflammation and hyperreactivity in non-antigen specific manner and independent of adaptive immunity [5]. In the T cell-mediated response, T cell receptor provides the antigen specific signal whereas costimulatory molecules provide the secondary signal to T cells resulting in an efficient T cell response. Unlike T cells, ILC2s lack any specific antigen receptor and receive activation signal mainly through cytokine stimuli, thus the expression of costimulatory molecules by ILC2s seems to be unnecessary. However, one of the hallmarks of ILC2 definition has been the expression of Inducible T cell COStimulator (ICOS) and the importance of ICOS is to the extent that it has been used as a means to specifically identify and deplete ILC2s in mice [6].

Recently, we found that ILC2s express not only ICOS but also the ICOS-L. To date, ICOS has been shown to be expressed solely by T cells whereas ICOS-L is expressed by B cell and antigen presenting cells but not T cells. ILC2s possess at least two unique features. First, they express, ICOS, a costimulatory molecule in the absence of any antigen specific receptor. Second, express ICOS-L and provide self-stimulation. We showed that genetic ablation of ICOS or blocking ICOS:ICOS-L interaction impairs phospho-STAT5 signaling, the cytokine production and the survival of ILC2s at the steady state and after IL-33 stimulation [7]. We further showed that our findings are translational as human ILC2s express both ICOS and ICOS-L and that ICOS:ICOS-L interaction is required for murine and human ILC2-mediated induction of airway inflammation and hyperreactivity in murine and humanized models [7]. We found evidence suggesting that *cis* interaction between ICOS and ICOS-L on the same ILC2 as well as the *trans* ICOS:ICOS-L between two adjacent ILC2s may occur. It remains to be studied, however, whether ILC2s have developed a unique self-stimulatory/regulatory pathway to delicately balance their number and function at the steady state and in the inflammatory conditions.

**Figure 1:**
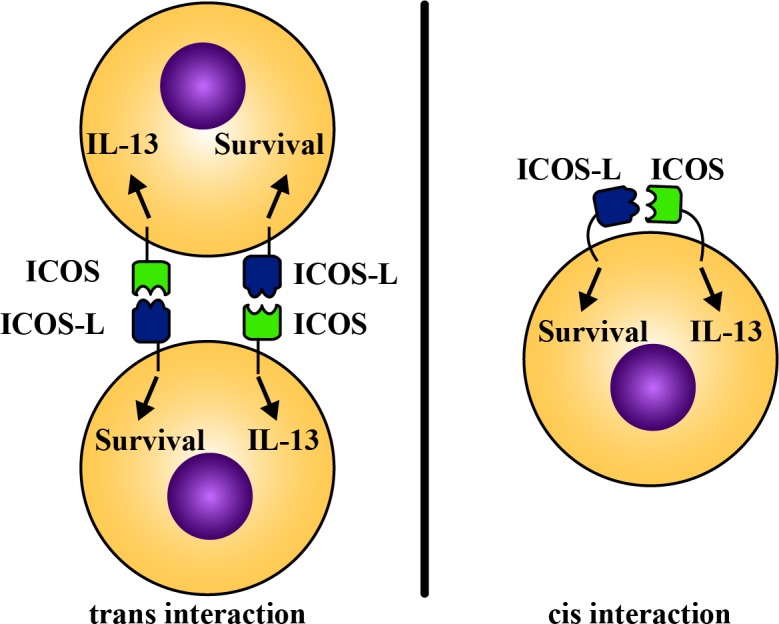
Signaling pathways downstream of ICOS and ICOS-L may differentially contribute to the cytokine production and survival of ILC2s

Since both ICOS and ICOS-L have functional cytoplasmic domains, signaling downstream of each molecule may differentially contribute to the cytokine production and / or survival of ILC2s (Figure 1). The cytoplasmic domain of ICOS is known to activate PI3K pathway and ultimately lead to cytokine production in T cells. Whether ICOS intracellular signaling follows a similar pathway in ILC2s is currently unknown. Expression of ICOS-L by ILC2s, leads to two conceivable explanations. The first is that ILC2s express ICOS-L solely to provide self-stimulation to ICOS and the signaling pathway downstream of ICOS-L is redundant for the survival and / or cytokine production by ILC2s. However, it is arguable that ICOS-L is abundantly expressed by B cells, dendritic cells and macrophages and the expression of ICOS-L by ILC2s may not seem necessary. The second and more likely explanation for the requirement of ICOS-L expression by ILC2s is that the signaling pathway downstream of ICOS-L is required for cytokine production or survival of ILC2s. ICOS-L signaling has been shown to play an important role for activation and IgG production by B cells. The contribution of ICOS-L signaling to cytokine production and / or survival of ILC2s remains to be studied.

Like any other cell, ILC2s play their roles in a team of immune and non-immune cells. It is interesting to understand how ILC2s interact with and influence other cells such as T cells, dendritic cells, lung epithelial cells and B cells. Cytokine production is the major known mechanism of communication between ILC2s with other cells. Nevertheless, expression of ICOS and ICOS-L by ILCs may offer a novel cell-cell contact mediated interplay of ILC2s with other cells. There is evidence indicating that a fraction of ILC2s express or acquire MHC-II and may be involved in antigen presentation to T cells [6]. In this context ICOS-L may offer an important costimulation to shape the T cell response e.g. towards Th2 cells. ILC2s may also play a major role in activating B cells and dendritic cells by providing ICOS costimulation. Further studies are required to address the role of ICOS and ICOS-L mediated costimulation in the interplay between ILC2s and other immune cells.

In summary, ICOS, ICOS-L expression by ILC2s offer a novel mechanism by which immune response to helminth infection and allergens are regulated. Targeting ICOS and ICOS-L pathways may open new windows to develop novel strategies for more specific and efficient therapeutics for asthma and allergic diseases as well as helminth infections.
